# The relationship between parental reflective function, cognitive emotion regulation and parental perception of the infant

**DOI:** 10.1192/j.eurpsy.2024.447

**Published:** 2024-08-27

**Authors:** K. Hunyadi, M. Miklósi, B. Szabó

**Affiliations:** ^1^Department of Developmental and Clinical Child Psychology, ELTE Eötvös Lóránd University Institute of Psychology; ^2^Department of Developmental and Clinical Child Psychology, ELTE Eötvös Lóránd Tudományegyetem University Institute of Psychology; ^3^Department of Clinical Psychology, Semmelweis University Faculty of Medicine; ^4^Heim Pál National Pediatric Institute, Centre of Mental Health; ^5^ELTE Eötvös Lóránd Tudományegyetem University, Doctoral School of Psychology, Budapest, Hungary

## Abstract

**Introduction:**

The literature indicates that parental reflective functioning (PRF) is crucial to a good parent-child relationship. Furthermore, genuine parental mentalizing also promotes adaptive emotion regulation in attachment relationships. However, no prior study assessed the relationship between parental mentalizing, emotion regulation and object relation in the early years.

**Objectives:**

We examined the relationship between PRF, cognitive emotion regulation and perception of the infant among parents of children up to five years old.

**Methods:**

In our cross-sectional, non-clinical study, 136 parents completed the Parental Reflective Functioning Questionnaire, the Cognitive Emotion Regulation Questionnaire and the Mother’s Object Relationship Scale - short form. In our 12 moderator models, we chose the subscales of the parental perception of the infant (invasiveness and warmth) as dependent variables, the subscales of adaptive and non-adaptive strategies of cognitive emotion regulation as independent variables, and the three subscales of PRF (pre-mentalization, interest and curiosity, certainty about mental states) as moderators.

**Results:**

Warmth had a positive, weak correlation with adaptive strategies (*r*(134) = 0.27, *p* < 0.007), with certainty in mental states (*r*(134) = 0.24, *p* < 0.007) and interest and curiosity (*r*(134) = 0.23, *p* < 0.007); the correlation between interest and curiosity and non-adaptive strategies was moderate and positive (*r*(134) = 0.32, *p* < 0.007). None of the subscales of PRF moderated the relationship between the subscales of emotion regulation and the perception of the infant. The use of adaptive emotion regulation strategies was more likely to affect the perception of warmth (B = 0.05 (*t* = 2.0584, *p* = 0.0415), B = 0.04 (*t* = 1.7887, *p* = 0.0760)), and the use of non-adaptive strategies was more likely to affect the perception of invasiveness (B = 0.08 (*t* = 2.1333, *p* = 0.0348), B = 0.09 (*t* = 2.3164, *p* = 0,0221).

**Conclusions:**

Our results suggest that cognitive emotion regulation plays a role in object relation; therefore, we recommend promoting adaptive cognitive emotion regulation strategies among mothers in the early years.

**Disclosure of Interest:**

None Declared

